# Proteomic analysis in the Dufour’s gland of Africanized *Apis mellifera* workers (Hymenoptera: Apidae)

**DOI:** 10.1371/journal.pone.0177415

**Published:** 2017-05-24

**Authors:** Aparecida das Dores Teixeira, Patricia D. Games, Benjamin B. Katz, John M. Tomich, José C. Zanuncio, José Eduardo Serrão

**Affiliations:** 1 Department of General Biology, Universidade Federal de Viçosa, Viçosa, Minas Gerais, Brazil; 2 Biotechnology Core Facility and Department of Biochemistry and Molecular Biophysics, Kansas State University, Manhattan, Kansas, United States of America; 3 Department of Entomology, Universidade Federal de Viçosa, Viçosa, Minas Gerais, Brazil; University of Salford, UNITED KINGDOM

## Abstract

The colony of eusocial bee *Apis mellifera* has a reproductive queen and sterile workers performing tasks such as brood care and foraging. Chemical communication plays a crucial role in the maintenance of sociability in bees with many compounds released by the exocrine glands. The Dufour’s gland is a non-paired gland associated with the sting apparatus with important functions in the communication between members of the colony, releasing volatile chemicals that influence workers roles and tasks. However, the protein content in this gland is not well studied. This study identified differentially expressed proteins in the Dufour’s glands of nurse and forager workers of *A*. *mellifera* through 2D-gel electrophoresis and mass spectrometry. A total of 131 spots showed different expression between nurse and forager bees, and 28 proteins were identified. The identified proteins were categorized into different functions groups including protein, carbohydrate, energy and lipid metabolisms, cytoskeleton-associated proteins, detoxification, homeostasis, cell communication, constitutive and allergen. This study provides new insights of the protein content in the Dufour’s gland contributing to a more complete understanding of the biological functions of this gland in honeybees.

## Introduction

Bees play a key role in agriculture and global ecosystems, pollinating plants in native areas as well as high value agricultural crops [1, 2]. They have different levels of social behavior, from solitary to eusocial, whose colony has a reproductive queen and thousands of sterile or semi-sterile workers, performing all other tasks for colony maintenance [3–5].

The honeybee *Apis mellifera* (Hymenoptera: Apidae) is a eusocial insect with a remarkable labor division [6]. Until two-three weeks of age, adult workers perform tasks inside the nest, including brood care and are named nurses [5]. After this period, the workers perform tasks out of colony including water, nectar, pollen and/or resin collection and are named foragers [5].

The social organization of the bees is maintained by a complex communication system that involves the pheromones release by various exocrine glands [6]. Among the exocrine gland of the bees, are those attached to the sting apparatus, including venom and Dufour’s glands [7]. The venom gland originates from glands associated with the ovipositor of ancestors Hymenoptera, whereas the Dufour’s gland is homologous to the lateral glands of other insects [8, 9].

A set of functions has been attributed to the Dufour’s gland secretion in different Hymenoptera, such as lining brood cells, nest building, larval feeding, in addition to its role in communication including hosts marker, nest and nest-mate recognition, fertility sign, trail [10], alarm [11] and sexual pheromones [12, 13]. Although the main compounds released by Dufour’s gland have been characterized as volatiles [10, 11], the proteins present in the gland remains poorly studied.

The objective of this study was to characterize those proteins expressed differentially in the Dufour’s gland of nurse and forager workers of *A*. *mellifera*, contributing with new data for the comprehension of the biological functions of this gland in the labor division.

## Materials and methods

### Biological samples

Africanized *Apis mellifera* were collected from Central Apiary of the Universidade Federal de Viçosa, Viçosa, Minas Gerais, Brazil (20° 45’ S 42° 52’ W). For this study, we analyzed 400 workers randomly collected from six colonies and in different dates. Two hundred nurse workers were collected in the brood areas inside the nest and 200 forager ones were collected in the nest entrance with pollen grains loaded in their hind legs. Nurse and forager workers were cryo-anesthetized, dissected and their Dufour’s glands were removed and stored in 1% (v/v) aqueous solution of protease inhibitors (Sigma P2714) at -20°C until use.

According Brazilian laws there is no need for ethical requirements for studies on invertebrates.

### Protein extraction

The samples of Dufour’s glands were dried using a Speed Vac concentrator (Concentrator Plus 5305, Eppendorf, Hamburg, Germany) and resuspended in 100 μL of lysis buffer (7 M urea, 2 M thiourea, 4% (w/v) 3-[(3-Cholamidopropyl) dimethylammonio]-1-propanesulfonate (CHAPS), and 40 mM dithiothreitol (DTT). Then, the samples immersed in an ice bath were submitted to successive ultrasound pulses (2 min) centrifuged at 15,000 x g for 30 minutes at 4°C and the supernatant collected. Total protein concentration was determined according to Bradford assay [14] using BSA as a standard.

### Two-dimensional electrophoresis and image acquisition

The samples were loaded onto immobilized pH gradient (IPG) strips (linear pH 3–10, 7 cm, GE Healthcare, USA). The strips were rehydrated for 12 h in 125 μL of rehydration solution (DeStreak—GE Healthcare, USA) containing 150 μg protein, 2% (v/v) ampholyte pH 3–10 (GE Healthcare) and 40 mM DTT. Isoelectric focusing (IEF) was performed in Ettan IPGphor III apparatus (GE Healthcare) at 20°C, with a maximum current of 50 mA/strip for approximately 14 h using the following program: 12 h at 300 V, 1000 V for 30 min, 5000 V for 80 min and 5000 V for 25 min. The IPG strips were first equilibrated in 5 mL equilibration buffer I [75 mM Tris-HCl, pH 8.8, 6 M urea, 25.5% (v/v) glycerol, 2% (w/v) sodium dodecyl sulfate (SDS), 0.002% (w/v) bromophenol blue and 1% (w/v) DTT] for 15 min and later in 5 mL equilibration buffer II [75 mM Tris-HCl, pH 8.8, 6 M urea, 25.5% (v/v) glycerol, 2% (w/v) sodium dodecyl sulfate (SDS), 0.002% (w/v) bromophenol blue and 2.5% (w/v) iodoacetamide] for 15 min. After equilibration, the strips were transferred to an SDS-polyacrylamide, 14% separating gel in Mini-PROTEAN Tetra cell (Bio-Rad, USA). The electrophoresis was performed at 100 V for 40 min and 60 V until the dye front reached the bottom of the gel. Gels were fixed in 40% (v/v) methanol with 10% (v/v) acetic acid for 30 min, washed in water, and stained with staining solution [0.8% (v/v) phosphoric acid, 20% (v/v) methanol, 8% (w/v) ammonium sulfate and 0.08% (w/v) Coomassie Brilliant Blue G-250 for 36 h. After this time, the gels were stored in solution containing 5% (v/v) acetic acid.

For each nurse or forager workers were carried out three gels, representing a triplicate.

The gels were digitized with Image Scanner III (GE Healthcare) at 16 bit and 600 dpi resolution. The images were analyzed using Image Master 2D Platinum 7.0 software (GE Healthcare, USA). ANOVA was carried out a threshold of p ≤ 0.05, and those spots with ≥ 1.2-fold change in average spot volume between nurse and forager workers were used for the mass spectrometry analyses.

### In-gel extraction and trypsin digestion of proteins for MALDI analysis

The differentially expressed spots in the Dufour’s gland of *A*. *mellifera* nurse and forager workers were manually excised from each gel and transferred to 0.6 mL microcentrifuge tubes containing solution of 50% (v/v) acetonitrile (ACN) and 25 mM ammonium bicarbonate (NH_4_HCO_3_). The samples were washed 1–4 times in this last solution until the gel pieces become translucent white. The gel slices were dehydrated for 5 min with 100% (v/v) ACN twice and dried at Speed Vac. Dried gel pieces were reduced with 65 mM DTT in 100 mM NH_4_HCO_3_ for 30 min at 56°C and alkylated with 200 mM iodoacetamide for 30 min in the dark at room temperature. The gel pieces were incubated with 100 mM NH_4_HCO_3_ and rehydrated with 100% (v/v) ACN for 10 min and dried using Speed Vac. Then the gel slices were digested in solution containing 40 mM NH_4_HCO_3_, 10% (v/v) ACN and 25 ng μL^-1^ trypsin (Trypsin Gold, Mass Spectrometry Grade, Promega, USA) overnight at 37°C. Peptides were extracted from the gel slices by incubating them with 5% (v/v) formic acid, 50% (v/v) ACN for 15 min. After incubation, gel slices were sonicated, and then the peptide solutions were transferred to a clean tube and dried in the Speed Vac to a final volume of 10 μL. The samples were desalted using a Zip-Tip C18 cartridge (Millipore, USA), dried in the Speed Vac and resuspended in 0.1% (v/v) aqueous trifluoroacetic acid (TFA) for MALDI mass analysis.

### Mass spectrometry and bioinformatics analyses

Desalted peptides samples from Dufour’s glands of both nurse and forager workers were mixed with α-cyano-4-hydroxycinnamic acid matrix [10 mg mL^-1^ in 50% (v/v) ACN, 0.1% (v/v) TFA] prepared in 1:1 ratio and the resulting 2 μL was spotted onto the MALDI plate MTP Anchor Chip TM 600/384 TF (Bruker Daltonics, Alemanha). The mass spectrometry (MS) analyses were performed on Ultraflex III MALDI TOF/TOF mass spectrometer (Bruker Daltonics, Germany). Spectra were acquired in the positive-ion reflectron mode with a mass range at 66.7 Hz with a 1000 laser shots added per spectrum. The MS/MS spectra were acquired in LIFT mode and 3000 laser shots. MS and MS/MS spectra were analyzed using FlexAnalysis 3.3 software (Bruker Daltonics, Germany) and calibrated internally with auto-proteolysis peptides of trypsin. All known contaminants (trypsin autoproteolysis and known keratin peaks) were excluded during the process. MS/MS spectra were processing using Sort Neaten Assign and Place (SNAP). Spectra were searched against the *A*. *mellifera* database (downloaded from uniprot.org, last update April 13, 2015) using the computer program Mascot (Mascot Daemon). Search parameters were as follows: taxonomy, other Metazoa; trypsin cleavage; allow up to one missed cleavage; peptide mass tolerance for MS, 0.5 Da; peptide mass tolerance for MS/MS, 0.2; fixed modification, carbamidomethyl (C); variable modification, oxidation (M). After identification by Mascot, the proteins were validated using Scaffold v.4.0 software. Peptide and Protein Prophet algorithms were used to establish the peptide and protein threshold at 90% respectively, with at least one unique peptide matches required in each sample. Proteins identified and validated were then analyzed using Uniprot database (http://www.uniprot.org/) to suggest probable function. Further, all the significant proteins were analyzed using various bioinformatics programme includes CELLO v.2.5 subcellular localization predictor (http://cello.life.nctu.edu.tw/), SIGNAL P v.4.1 signal peptide predictor (http://www.cbs.dtu.dk/services/SignalP/) and STRING v.10.0 database protein-protein interaction predictor (http://www.string-db.org/).

## Results

### Comparative 2D gel electrophoresis

**Fig 1:**
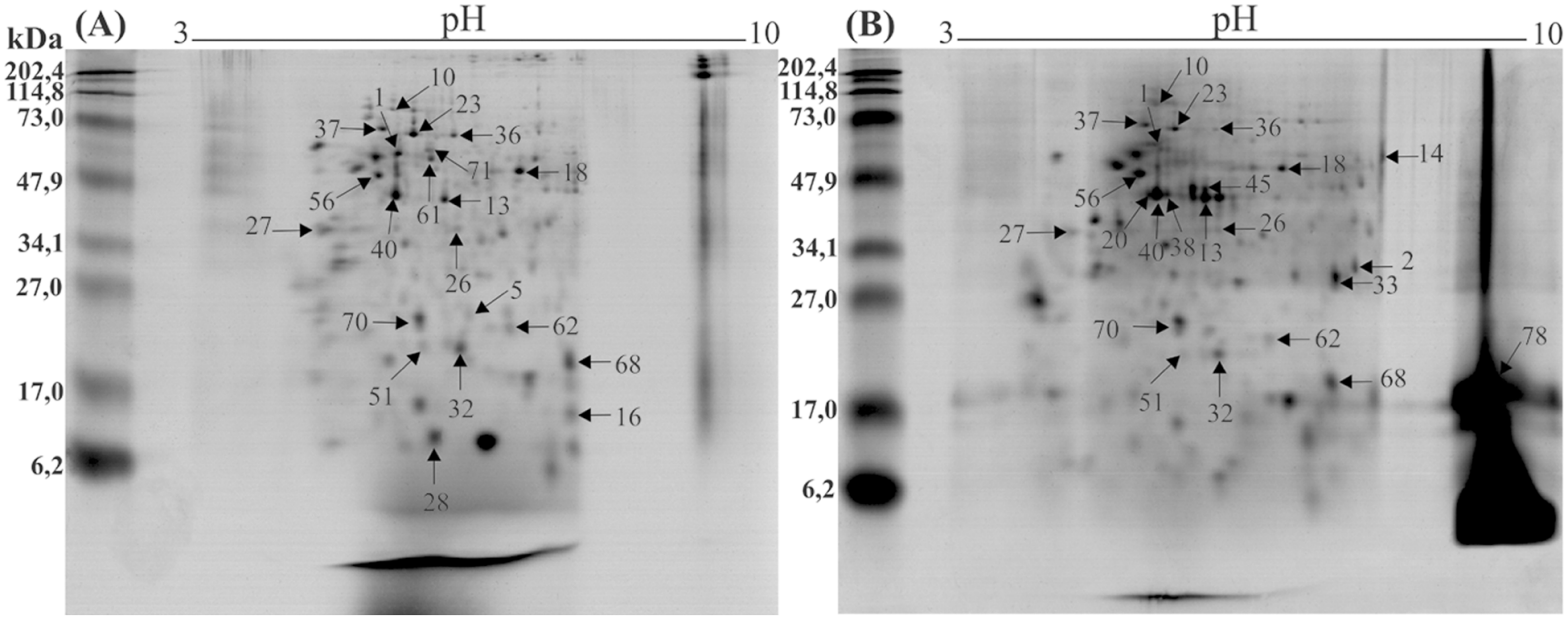
Representative 2-D electrophoresis proteins profile from Dufour’s gland of *Apis mellifera* nurse (A) and forager (B) workers. All identified proteins by MALDI-TOF/TOF are numbered and spot numbers correspond to Table 1.

The 2D-electrophoresis profiles obtained from the Dufour’s gland of nurse and forager workers of *A*. *mellifera* revealed 279 spots (Fig 1). The set of ANOVA (p < 0.05) analyses detected 131 spots showing differential expression between nurse and forager workers (see S1 Table).

### Mass spectrometry

A total of 80 protein spots from Dufour’s glands of *A*. *mellifera* workers were excised for digestion and mass spectrometry analyses. The MS/MS analyses resulted in the identification of 28 spots with 24 different proteins (Fig 1A and 1B, Table 1).

**Table 1:** List of identified proteins spots in Dufour’s glands of *Apis mellifera* nurse and forager workers.

Spot number^a^	Accession number	Protein name	MM (Da)/pI Experimental	MM (Da)/pI Theorical	ANOVA^b^	Score^c^
01	V9II55_APICE	Heat Shock protein (HSP60)	57139/5.61	60546/5.64	0.000059	82
02	A0A088A4K0_APIME	Phosphoglycerate mutase	31688/7.77	35396/9.36	0.000068	156
05	J7F0F7_APICC	Ferritin	23473/6.42	25718/5.9	0.00037	79
10	V9IJ68_APICE	Transitional endoplasmic reticulum ATPase	86885/5.57	89467/5.18	0.0012524	74
13	A0A088ARZ8_APIME	Arginine kinase	42360/6.16	43726/6.11	0.0013432	414
14	V9II98_APICE	Chitinase-like protein Idgf4	56395/8.13	48921/8.06	0.0014878	162
16	Q76LA4_APIME	Fatty acid binding protein	10636/7.66	15140/6.37	0.0021074	61
18	A0A088AST9_APIME	Enolase	49088/7.10	39821/6.54	0.0024789	336
20	V9IE14_APICE	Actin-42A	45085/5.27	42121/5.3	0.004775	169
23	V9IEZ2_APICE	Heat shock protein (HSP70)	65541/5.80	71383/5.43	0.0052428	504
26	A0A088ATC7_APIME	Proteasome subunit alpha type	36131/6.31	31250/5.78	0.0063259	56
27	A0A088AJH1_APIME	Annexin	35612/4.53	36088/4.6	0.0072667	108
28	Q1W641_APIME	Odorant Binding Protein 13 (OBP13)	7873/5.95	15494/6.37	0.0090299	123
32	G0WRL1_APICC	thioredoxin peroxidase 1(Tpx-1)	20221/5.82	22065/6.31	0.0111726	106
33	L0HT99_APICE	Actin (Fragment)	30141/7.54	31458/4.94	0.01178530	41
36	V9IK10_APICE	Stress-70 protein (HSP70)	65221/6.30	75642/6.38	0.0132579	114
37	V9IGA1_APICE	Glucose-regulated protein (HSP70)	68495/5.44	72878/5.29	0.0136561	298
38	V9IE14_APICE	Actin-42A	44934/5.51	42121/5.3	0.0138284	94
40	V9IE14_APICE	Actin-42A	43919/5.57	42121/5.3	0.0230893	253
45	ACPH1_APIME	Venom acid phosphatase Acph-1	46005/5.99	45588/5.63	0.025984	135
51	V9IJF3_APICE	Glutathione S-transferase	22562/5.81	22997/5.49	0.0312186	245
56	A0A088AMB8_APIME	ATP synthase subunit beta	48372/5.37	55096/5.25	0.0384374	471
61	A0A088A7Y2_APIME	Protein disulfide-isomerase	55213/6.05	6220/5.57	0.0423643	157
62	V9IG54_APICE	Protein lethal(2)essential for life (HSP20)	21819/6.93	21882/6.32	0.0447684	86
68	I1VC86_APIME	Phospholipase A2	18400/7.66	19585/7.05	0.0200282	50
70	V9IJF3_APICE	Glutathione S-transferase	23544/5.80	22997/5.49	0.0152591	141
71	A0A088A9C4_APIME	Carboxylic ester hydrolase	59133/5.97	59330/5.55	0.0173495	139
78	I1VC86_APIME	Phospholipase A2	18302/9.30	19585/7.05	0.0219103	107

^a^Spot numbers as in 2D reference gel (see Fig 1).

^b^ANOVA values from Image Master software.

^c^Mascot scores; Individual ions scores > 27 indicate identity or extensive homology (p<0.05).

**Fig 2:**
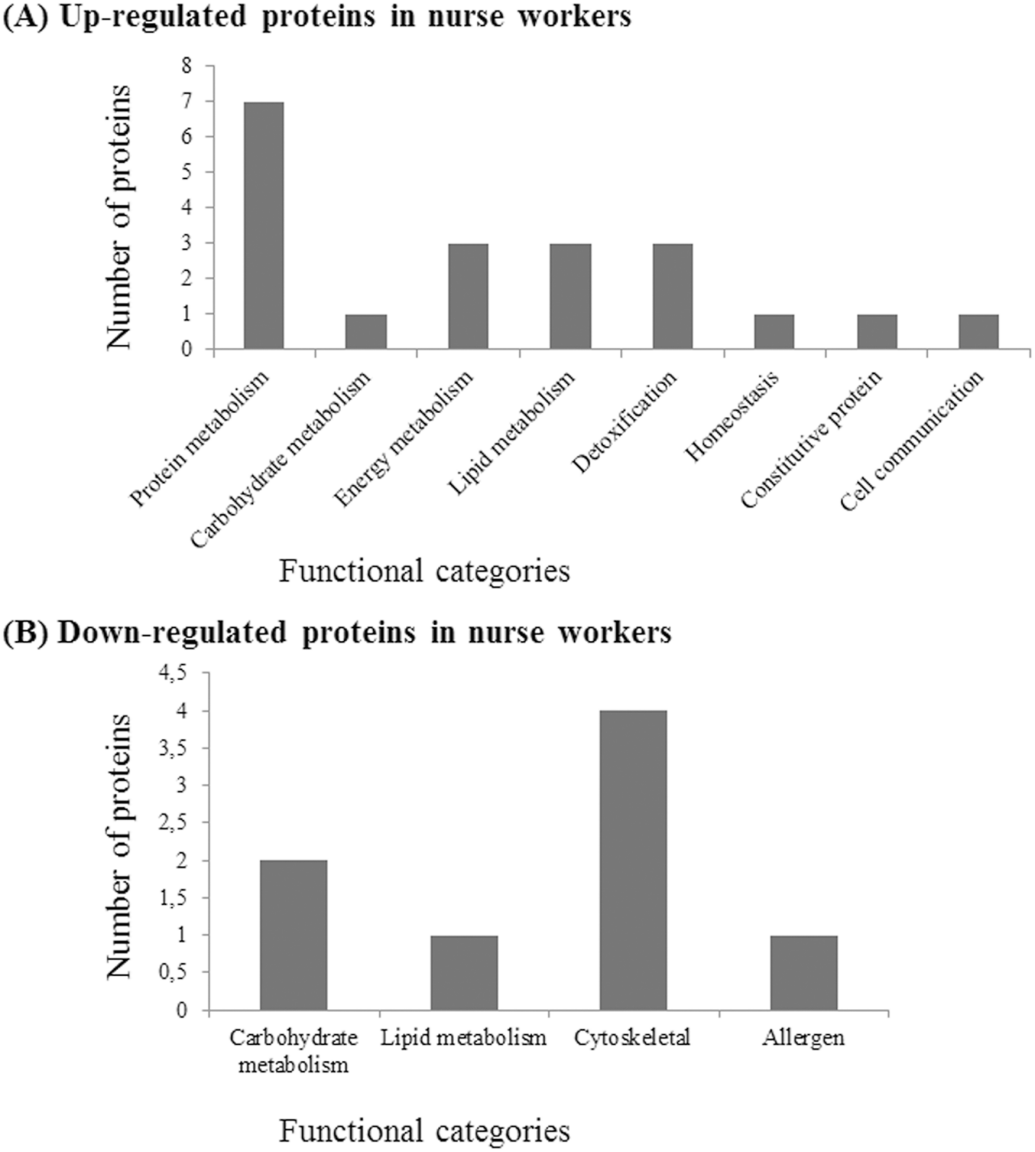
Identified proteins from Dufour’s gland of Apis mellifera workers. Up-regulated (A) and down-regulated (B) proteins in nurse workers. Classification into functional categories was obtained from the Uniprot database.

Proteins in the up-regulated group in nurse workers (a total of 20 proteins identified) included proteins that were more expressed higher in nurse workers compared to forager bees (c.a. 15 proteins) as well as those proteins that were present only in nurse workers (c.a. 5 proteins) (Fig 2, Table 2). These proteins belong to different functional categories playing roles in the metabolisms of protein (spot 1, 23, 26, 36, 37, 61 and 62), carbohydrates (spot 18), energy (spots 10, 13 and 56), and lipid (spots 16, 68 and 71), detoxification (spots 32, 51 and 70), homeostasis (spot 5), constituent protein (spot 27) and cell communication (spot 28) (Fig 2A, Table 2).

**Table 2:** List of identified proteins spots in Dufour’s glands of *Apis mellifera* workers with their fold expression values, functional categories, subcellular localization and signal peptide analyses.

Spots number^a^	Protein name	Expression	Fold change^b^	Functional categories	Subcellular localization	Signal peptide
01	Heat Shock protein (HSP60)	Up	16.49	Protein metabolism	Cytoplasmic	No
23	Heat shock protein (HSP70)	Up	7.41	Protein metabolism	Cytoplasmic	No
26	Proteasome subunit alpha type	Up	5.21	Protein metabolism	Cytoplasmic/nucleus	No
36	Stress-70 protein (HSP70)	Up	9.92	Protein metabolism	Mitochondria	No
37	Glucose-regulated protein (HSP70)	Up	2.93	Protein metabolism	Endoplasmic reticulum	No
61	Protein disulfide-isomerase	Up	∞	Protein metabolism	Endoplasmic reticulum	Yes
62	Protein lethal(2)essential for life (HSP20)	Up	2.89	Protein metabolism	Mitochondria / Cytoplasmic	No
02	Phosphoglycerate mutase	Down	∞	Carbohydrate metabolism	Cytoplasmic	No
14	Chitinase-like protein Idgf4	Down	∞	Carbohydrate metabolism	Extracellular	Yes
18	Enolase	Up	4.70	Carbohydrate metabolism	Cytoplasmic	No
10	Transitional endoplasmic reticulum ATPase	Up	2.46	Energy metabolism	Endoplasmic reticulum	No
13	Arginine kinase	Up	1.84	Energy metabolism	Cytoplasmic	No
56	ATP synthase subunit beta	Up	1.54	Energy metabolism	Mitochondria	No
16	Fatty acid binding protein	Up	∞	Lipid metabolism	Cytoplasmic	No
68	Phospholipase A2	Up	2.62	Lipid metabolism	Extracellular	Yes
71	Carboxylic ester hydrolase	Up	∞	Lipid metabolism	Cytoplasmic	No
78	Phospholipase A2	Down	∞	Lipid metabolism	Extracellular	Yes
20	Actin-42A	Down	∞	Cytoskeletal	Cytoplasmic	No
33	Actin (Fragment)	Down	∞	Cytoskeletal	Cytoplasmic	No
38	Actin-42A	Down	∞	Cytoskeletal	Cytoplasmic	No
40	Actin-42A	Down	3.49	Cytoskeletal	Cytoplasmic	No
32	Thioredoxin peroxidase 1 (TPX protein)	Up	3.23	Detoxification	Cytoplasmic	No
51	Glutathione S-transferase	Up	1.67	Detoxification	Cytoplasmic	No
70	Glutathione S-transferase	Up	4.79	Detoxification	Cytoplasmic	No
05	Ferritin	Up	∞	Homeostasis	Extracellular	Yes
27	Annexin	Up	3.16	Constitutive protein	Cytoplasmic	No
28	Odorant Binding Protein 13 (OBP13)	Up	∞	Cell communication	Extracellular	Yes
45	Venom acid phosphatase Acph-1	Down	∞	Allergen	Extracellular	Yes

^a^Spot numbers as in 2D reference gel (see Fig 1).

^b^Fold change is the ratio between average normalized volume of each protein in nurse workers that of the corresponding spot in forager ones gel. ∞ = means that spots of the all replicates are presents only in nurse or forager workers.

Proteins down-regulated group in nurse workers (a total of eight proteins identified), included those proteins with lower expression levels in the nurse workers in relation to forager ones (c.a. one protein) as well as those different proteins presents only in forager workers (c.a. five proteins) (Table 2). These proteins have been classified into carbohydrate (spot 2 and 14) and lipid (78) metabolisms, cytoskeleton-associated proteins (spots 20, 33, 38 and 40) and allergen (spot 45) (Fig 2B, Table 2).

### Subcellular localization and signal peptide

**Fig 3:**
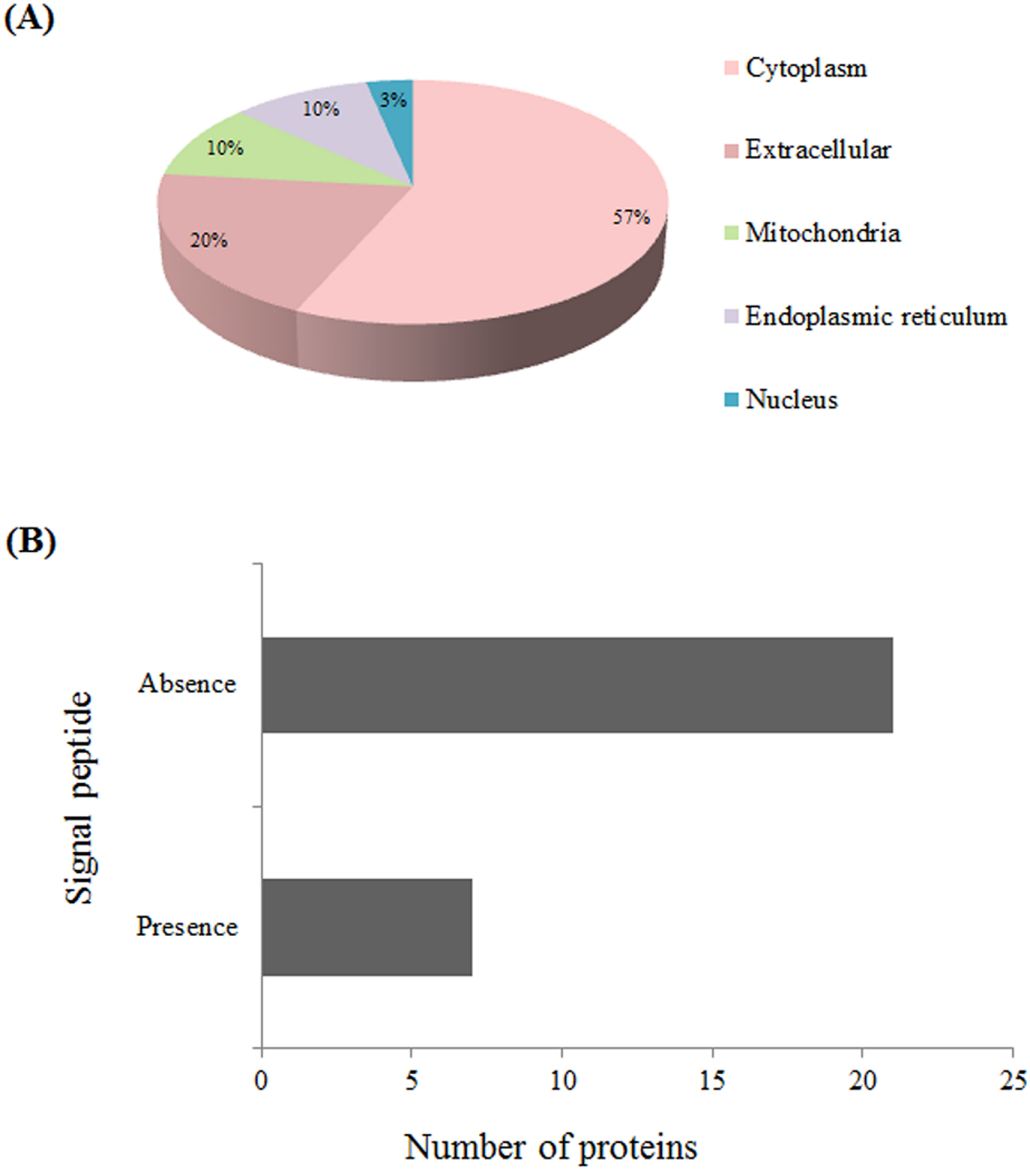
Identified proteins from Dufour’s gland of *Apis mellifera* workers. (A) Classification into subcellular localizations obtained from the CELLO v.2.5 subcellular localization predictor. (B) Peptide signal presence obtained from the SIGNAL P v.4.1.

Data on subcellular localization and the presence of signal peptide in the identified proteins were evaluated taking into account secretory function of Dufour’s gland (Fig 3, Table 2). Most of the identified proteins (c.a. 57%) showed cytoplasmic subcellular localization (Fig 3A) and a small group (c.a. 25%) of the proteins contained signal peptides (Fig 3B).

### Interaction of expressed proteins

**Fig 4:**
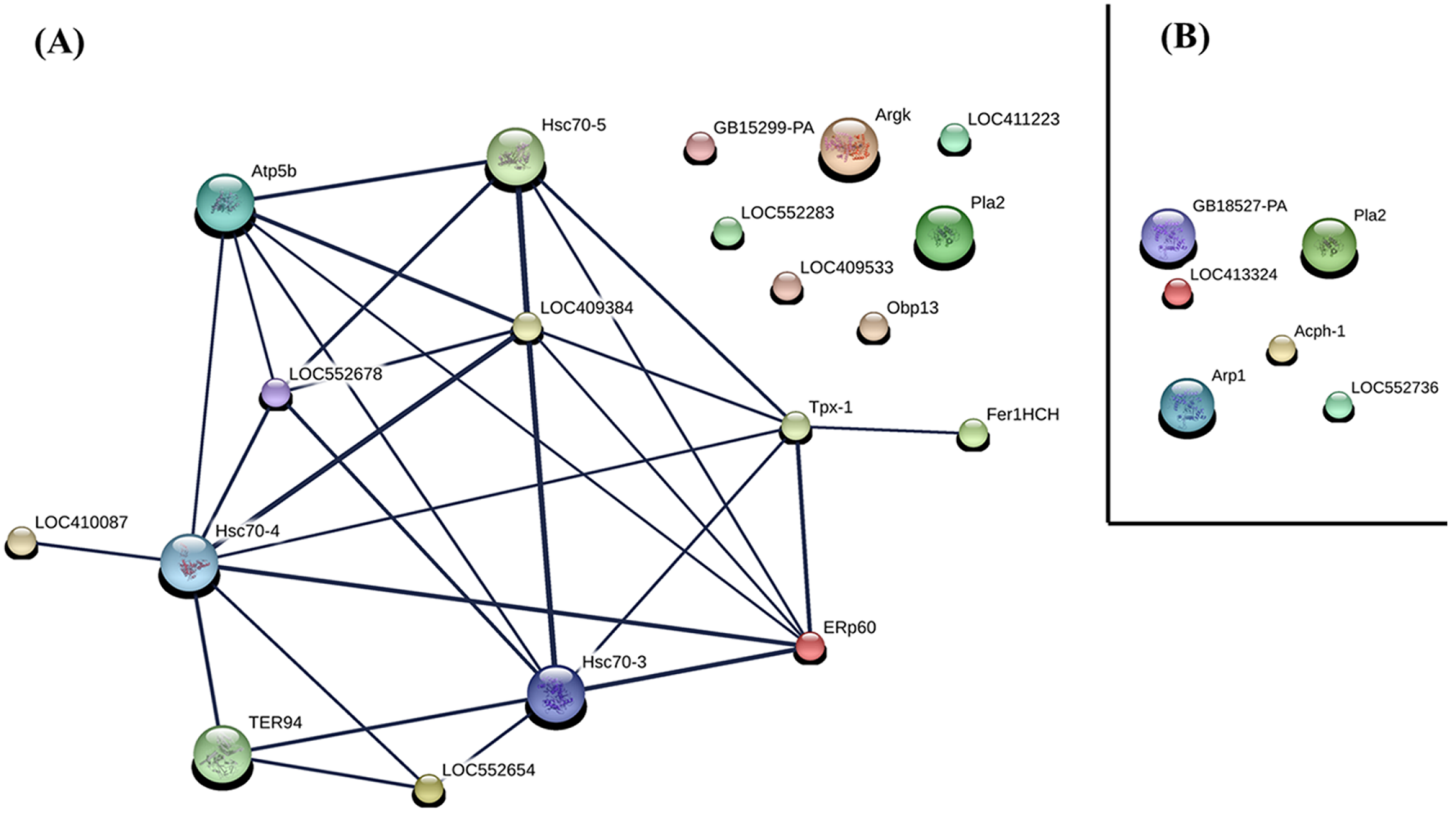
Protein-protein interactions of the identified proteins from Dufour’s gland of Apis mellifera workers. Up-regulated (A) and down-regulated (B) proteins in nurse workers in comparison with forager ones. Proteins were analyzed using the STRING database. The numbers correspond to the protein accession numbers in the Uniprot database and are described in the text. The interactions networks are shown in the confidence view where thicker and thinner lines represent the stronger and weaker interactions, respectively.

Protein-protein predicted functional interactions were assessed to obtain additional information about the functions of the protein of the Dufour’s gland in *A*. *mellifera* workers (Fig 4).

Most of the up-regulated proteins of Dufour’s gland of nurse workers showed functional interactions among them: heat shock protein (LOC409384), transitional endoplasmic reticulum ATPase (TER94), enolase (LOC552678), heat shock protein (Hsc70-4), proteasome subunit alpha type (LOC552654), thioredoxin peroxidase 1 (Tpx-1), stress-70 protein (Hsc70-5), glucose-regulated protein (Hsc70-3), ATP synthase subunit beta (Atp5b), protein lethal (2) essential for life (LOC410087), protein disulfide-isomerase (ERp60) and ferritin (Fer1HCH) (Fig 4A).

The heat shock protein (LOC409384) exhibited functional interactions with most of the protein interaction network, but there was a predominance of stronger interactions with the glucose-regulated protein (Hsc70-3), heat shock protein (Hsc 70–4) and stress protein-70 (Hsc70-5), all them a family of heat shock proteins (HSPs), besides of interaction with ATP synthase subunit beta (Atp5b) (Fig 4A).

The thioredoxin peroxidase 1 (Tpx-1) exhibited strong functional interactions with the protein disulfide isomerase (ERp60), which in turn showed interactions with heat shock proteins (Hsc70-3 and Hsc70-4) (Fig 4A). In addition, Tpx-1 interacted weakly with ferritin (Fer1HCH).

All down-regulated proteins in Dufour’s gland of nurse workers including chitinase-like protein Idgf4-like (LOC413324), phosphoglycerate mutase (LOC552736), actin fragment (GB18527-PA), actin 42A (Arp 1), phospholipase A2 (Pla2) and venom acid phosphatase Acph-1 (Acph-1) showed no functional interactions among themselves (Fig 4B).

## Discussion

All of the proteins identified that exhibited activity in protein metabolism showed different levels in the Dufour’s gland of nurse workers of *A*. *mellifera* such as heat shock proteins (HSPs) superfamily: heat shock protein (HSP60), heat shock protein (HSP70), stress-70 protein (HSP70), glucose-regulated protein (HSP70) and protein lethal (2) essential for life (HSP20). On the other hand, protein disulfide isomerase was expressed solely in the Dufour’s gland of nurse workers. Molecular chaperones and proteases are required to the normal functionality of proteins in a living cell showing synthesis/degradation activities [15], controlling the balance between native folded functional proteins and aggregation-prone misfolded or invalidated proteins [16]. To this effect, proteins involved in configuration of nascent peptide chains are expressed as heat shock proteins (HSPs) and protein disulfide-isomerase. In addition, proteins with degradative function [17] such as the proteasome subunit alpha type are also required to keep this balance.

In honeybees, HSPs are induced at high temperatures [18], under pathogenic infections conditions [19, 20] and processes of embryogenesis [21].

The protein lethal (2) essential for life in *A*. *mellifera* is up-regulated in the brain of forager workers [22, 23] and seems play some role during bee aging. However, the function of this protein in the Dufour’s gland of *A*. *mellifera* workers needs further studies.

All proteins discussed above are functionally related among themselves (see Fig 4A) and seem play some role in protein metabolism of Dufour’s gland. Although most of the secreted products released by Dufour’s gland of the bees are lipids [24, 25], these proteins may be involved in the metabolism of other proteins, with their metabolic products used in the production of hydrocarbons, alcohols and esters, which have been reported in isolated secretions of this gland from nurse workers [26–29].

Within proteins of the carbohydrate metabolism family, phosphoglycerate mutase and chitinase-like protein Idgf4 were found only in the Dufour’s gland of forager workers, whereas enolase was more expressed in nurse ones. Both phosphoglycerate mutase and enolase are involved in the glucose oxidation [30, 31] which is the major energy substrate for various tissues of bees [32, 33]. The chitinase-like protein Idgf4 is an imaginal disc growth factors, related to chitinase, but unlike these, chitinase-like protein Idgf4 has not hydrolase activity [34]. In *A*. *mellifera*, Idgf4 has been reported to act in the larval development [35, 36] and it seems to be released and transported to target tissues via insect hemolymph [37].

The abundance of these energy conversion proteins from carbohydrate in the Dufour’s glands of forager (phosphoglycerate mutase) and nurse (enolase) workers, suggests that this gland in both workers have an important function in cellular processes with high energy consumption, showing that Dufour’s gland remains active throughout the bee lifespan [38]. The function of the chitinase-like protein Idgf4 found only in the Dufour’s gland of forager workers remains unknown.

Proteins of energy metabolism as arginine kinase, ATP synthase subunit beta and transitional endoplasmic reticulum ATPase were more highly expressed in Dufour’s gland of nurse workers. Arginine kinase has been reported to be both a cytoplasmic and mitochondrial protein in arthropods [39, 40] and is up-regulated in *Apis cerana cerana* workers under biotic and abiotic stresses [41] while ATP synthase subunit beta, in addition to its role energy production, has been reported to act as a lipophorin binding protein, the main lipoprotein in the circulation of the insects [42], which cycles among peripheral tissues to exchange its lipid cargo at the plasma membrane of target cells [43]. The transitional endoplasmic reticulum ATPase, in turn, has been characterized as key mediator involved in ER-associated degradation (ERAD), a major quality control process in the protein secretary pathway [44, 45].

These enzymes may function in high energy reactions required for the transport of substances from the hemolymph, which mediates chemical exchanges among tissues and organs, besides to serving in water storage [46, 47] or even modifying compounds in the gland cells prior their release into the gland lumen. The morphology of cells in the Dufour’s gland of queens and workers honeybees, suggest that they are able to uptake substances from hemolymph, as circulating fatty acids [25, 48–51], which may be used as substrates in the production of hydrocarbons and esters found in the Dufour’s gland secretions [26–29, 52, 53].

The presence of proteins involved in lipid metabolism include fatty acid binding protein and hydrolase carboxylic ester, present only in nurse workers, and phospholipase A2, present in both nurse as forager in Dufour’s gland of *A*. *mellifera* is logical because the principal products released by the gland are lipid-based compounds [24, 25, 52, 53].

Fatty acid binding proteins transfer fatty acids for cell membranes and mediate the effect of fatty acids on gene expression in different tissues [54] while carboxylic ester hydrolases catalyze the hydrolysis of an ester bond resulting in the formation of an alcohol and a carboxylic acid [55]. In addition, phospholipases A2 (Pla2s) catalyze the hydrolysis of the sn-2 acyl glycerophospholipd releasing free fatty acids and lysophospholipids [56]. All proteins described above seem be involved in the production of lipids compound and its precursors found in the Dufour’s gland secretion in honey bees [26–29, 52, 53].

A hypothetical model has been suggested for esters production in Dufour’s gland of queens and workers of *A*. *mellifera*, in which esters are synthesized by the elongation of fatty acids into long chain acyl groups, following by their conversion to alcohols and subsequent esterification by acyl-CoAs [57–59]. Eicosanol, an alarm pheromone [28, 60] from workers may be an intermediate in the secretory pathway of esters in the Dufour’s gland [59]. In the presence of the queen, this secretory pathway is stopped, and the workers produce only precursor esters, but in queenless colonies, this route proceeds to esters [59]. This may explain the increase in the expression of lipid metabolism proteins in Dufour’s gland of nurse workers that potentially may lay eggs in the absence of the queen [61, 62] since the secretion of this gland has been reported to be related to the reproductive status of eusocial bees [59].

The detoxification and oxidative stress proteins, thioredoxin peroxidase-1 (Tpx-1) and glutathione S-transferase (GST) were up-regulated in Dufour’s gland of nurse workers. These proteins are functionally related (see Fig 4A), degrading potentially harmful substances such as reactive oxygen species and acting in the detoxification of endogenous and xenobiotic compounds [63, 64]. They are also important in intercellular transport, horm one biosynthesis and insect resistance to insecticides [65, 66]. In adult worker bees, GSTs have been reported in the muscle, brain, fat body, epidermis, midgut, and fat body [67] and venom [68].

The up-regulation of these detoxification proteins in nurse workers, may be due to the higher metabolic activity of Dufour’s gland than in forager ones, because the cell metabolism results in the production of reactive oxygen species that are toxic for cells [69].

Ferritin was expressed only in the Dufour’s gland of nurse worker. This protein transport iron ions contributing to cell homeostasis [70]. Furthermore, it seems to play role in detoxification process in some insects [71], sharing biological functions with Tpx-1 (see Fig 4A). Studies on insect ferritins revealed that most insects have abundant ferritin in the hemolymph and in the secretory pathways of the cells [72]. Its high expression in Dufour’s gland of *A*. *mellifera* nurse workers is not clear.

Odorant binding protein OBP-13 plays important role in chemical communication in insects [73]. The genome of *A*. *mellifera* predict 21 OBPs (OBP 1–21) [74, 75], which are small water soluble molecules, binding reversibly to messenger molecules such as pheromones that are generally hydrophobic molecules with low solubility [76, 77]. Although OBPs are commonly expressed in sensory organ of insects such as gustatory and olfactory sensilla [77], they have been also reported in ovary, brain, tegument [74, 78], mandibular gland [79], hemolymph [37] and head salivary gland [80] of social bees. In the present work, OBP-13 was up-regulated in the Dufour’s gland of younger nurse workers similarly to that previously reported to *A*. *mellifera* which OBP-13 expression is caste and age regulated, with high expression in younger workers [81]. Its high expression in nurse workers may be associated with increased production of pheromones by the Dufour’s gland in these bees.

An intriguing finding is the presence of venom acid phosphatase Acph-1 in the Dufour’s gland of *A*. *mellifera* forager workers. This is an enzyme of bee venom [68, 82] that hydrolyzes phosphomonoesters at acidic pHs. The Acph-1 of bee venom is a potent allergen, triggering strong reactions in humans [83]. The presence of this protein in the Dufour’s gland of forager workers might be due a possible contamination from the venom gland, but during the removal of the Dufour’s gland there were no apparent disruptions of the venom reservoirs. Thus, this protein may have another function in these worker bees.

The chemical composition of Dufour’s gland secretion in insects has been identified mainly by gas chromatography and mass spectrometry, as being composed for saturated or unsaturated long chain hydrocarbons, although other compounds may also occur in this gland [10, 24, 52, 53, 84–86]. The principal enzymes for the hydrocarbons biosynthesis are fatty acid synthases, elongases, desaturases, reductases and P450 decarboxylase [12, 87]. In the honey bee *A*. *mellifera*, six desaturase and 10 elongases genes have been predict with some them expressed differentially in nurse and forager workers [88]. However, these hydrocarbon metabolism enzymes were not identified in this study, perhaps because as small they were not detected using a 2D gel/MALDI-TOF/TOF protocol or because we only analyzed those spots with significant difference in the two-dimensional electrophoresis suggesting that these enzymes are expressed equally in both the Dufour’s gland of nurse and forager workers. This result suggests that further studies are necessary to identify the complete profile of proteins of the Dufour’s gland of *A*. *mellifera*.

This is the first descriptive and comparative proteomic study to characterize proteins in the Dufour’s gland of *A*. *mellifera*. Our results showed differences in the quantity and types of proteins in the Dufour’s gland when nurse workers change to forager ones, suggesting that this gland has different roles according to bee age and social behavior. Despite its simple structural complexity, the Dufour’s gland of bees seem to have a great functional complexity, since until now, its major role has been reported as the production of lipid other than proteins. This study can be useful for the comprehension of some molecular mechanisms triggered the labor division in bees.

## Supporting information

S1 Table List of identified proteins spots in Dufour’s gland of *Apis mellifera* workers with Image Master. Up-regulated and down-regulated refer to proteins with higher and lower expression, respectively, in nurse worker in comparison with forager ones.
(PDF)
