# Supplementary material for: Proteomic analysis in the Dufour’s gland of Africanized Apis mellifera workers (Hymenoptera: Apidae)
Source: PLoS One. 2017 May 24;12(5):e0177415. doi: 10.1371/journal.pone.0177415 (PMC5443511; doi:10.1371/journal.pone.0177415)
Supplement: S1 Table — Up-regulated and down-regulated refer to proteins with higher and lower expression, respectively, in nurse worker in comparison with forager ones. (PDF) [file pone.0177415.s001.pdf]

S1 Table. List of identified proteins spots in Dufour's gland of *Apis mellifera* workers with Image Master. Up-regulated and down-regulated refer to proteins with higher and lower expression, respectively, in nurse worker in comparison with forager ones.

Up-regulated				Down-regulated			
**Spot[a]**	**pI**	**MM (Da)**	**ANOVA[b]**	**Spot[a]**	**pI**	**MM (Da)**	**ANOVA[b]**
1	5.61	57139	0.0000593	2	7.77	31688	0.0000678
3	7.23	32220	0.000272	6	7.96	53784	0.000523
4	6.61	7804	0.000368	9	7.40	21761	0.001216
5	6.42	23473	0.000371	11	3.67	18147	0.001314
7	6.16	65863	0.000585	14	8.13	56395	0.001488
8	6.32	46033	0.000838	15	6.06	23561	0.001839
10	5.57	86885	0.001252	19	3.93	22451	0.003225
12	7.69	6834	0.001318	20	5.27	45085	0.004775
13	6.17	42360	0.001343	24	7.47	77542	0.005843
16	7.67	10636	0.002107	25	6.14	42293	0.006233
17	6.91	23616	0.002322	30	8.18	127894	0.009319
18	7.11	49088	0.002479	31	7.94	29348	0.010489
21	5.28	76470	0.004788	33	7.55	30141	0.011785
22	5.46	71935	0.004995	34	6.15	40483	0.012846
23	5.80	65541	0.005243	38	5.52	44934	0.013828
26	6.31	36131	0.006326	40	5.57	43919	0.023089
27	4.53	35612	0.007267	41	5.85	10490	0.023666
28	5.96	7873	0.00903	44	8.16	303471	0.024914
29	6.17	20782	0.009129	45	5.99	46005	0.025984
32	6.31	20221	0.011173	47	5.99	40212	0.027086
35	6.69	22019	0.012923	48	5.34	62003	0.028368
36	6.31	65221	0.013258	54	5.85	30242	0.037502
37	5.45	68495	0.013656	55	5.50	99718	0.037525
39	6.99	54943	0.013994	66	8.15	36597	0.047402
42	6.55	46535	0.024275	72	8.06	18936	0.018638
43	4.97	66511	0.024425	73	7.75	39942	0.019149
46	5.16	21952	0.026953	75	6.69	85750	0.021027
49	5.25	92077	0.028667	76	4.92	34446	0.02108
50	7.43	47555	0.030707	77	5.68	14063	0.021637
51	5.82	22562	0.031219	78	9.30	18302	0.02191
52	6.78	50059	0.032374	79	10.05	18095	0.022583
53	5.42	42207	0.036354	84	7.57	8030	0.031091
56	5.37	48372	0.038437	85	5.51	8753	0.022703
57	5.28	110871	0.040862	86	7.39	15596	0.042534
58	4.43	23048	0.041834	87	4.06	25008	0.025184

59	8.04	17689	0.041968	88	5.48	25875	0.031942
60	7.15	13993	0.042161	89	5.28	32653	0.007584
61	6.01	55213	0.042364	90	5.34	35030	0.001622
62	6.93	21819	0.044768	91	4.58	36597	0.000476
63	4.76	38006	0.046459	92	7.19	44184	0.014615
64	5.79	12922	0.046859	93	6.52	44036	0.00161
65	5.81	83911	0.046915	94	7.48	46315	0.009
67	4.48	49329	0.048343	95	6.01	54069	0.001296
68	7.67	18400	0.020028	96	5.73	54930	0.048578
69	5.99	108326	0.015139	97	5.84	56099	0.009434
70	5.80	23544	0.015259	98	7.17	57293	0.02415
71	5.98	59133	0.01735	99	6.92	58206	0.004027
74	4.47	59715	0.019161	100	5.89	68891	0.018703
80	5.43	19201	0.022794	101	4.14	76766	0.022683
81	5.27	11117	0.015868	102	5.61	85750	0.006098
82	5.84	53877	0.001369	103	6.25	96753	0.006307
83	6.40	83911	0.015666	129	7.82	47260	0.002082
104	7.70	42513	0.028292	130	5.61	30242	0.023893
105	6.50	49571	0.005058	131	7.61	46315	0.028208
106	6.36	21490	0.00133				
107	5.62	25405	0.001744				
108	6.97	28009	0.0000231				
109	6.57	28480	0.003329				
110	5.46	32006	0.043583				
111	6.41	33648	0.00000966				
112	5.05	36000	0.033912				
113	7.31	36261	0.012412				
114	7.46	36790	0.009538				
115	6.73	41004	0.024198				
116	6.77	42513	0.001255				
117	7.80	44238	0.001479				
118	5.27	47042	0.002517				
119	5.70	47042	0.031576				
120	6.18	51301	0.006572				
121	6.08	50801	0.016263				
122	6.08	64269	0.001688				
123	5.59	69851	0.006237				
124	6.55	82943	0.000327				
125	5.82	108326	0.038003				
126	5.58	125265	0.005639				
127	5.81	122562	0.034573				
128	6.18	133735	0.02066				

[a] Spots showing differential expression between nurse and forager workers. [b]ANOVA values from Image Master software ($p<0.05$).
